# Knowledge of cytogenetic analysis for synovial sarcoma in sarcomatoid variant renal cell carcinoma

**DOI:** 10.1002/ccr3.5052

**Published:** 2021-11-07

**Authors:** Vishal Farid Raza, Dawood Arshad, Khalid Irshad, Khalid Javeed Khan

**Affiliations:** ^1^ Fatima Jinnah Medical University Lahore Pakistan

**Keywords:** renal cell carcinoma, sarcomatoid, surgery, t (X;18)

## Abstract

Sarcomatoid change in a renal tumor should undergo cytogenetic analysis of t(x;18) to prevent a missed diagnosis of synovial sarcoma. Surgeons should be vigilant regarding pathological correlation.

## Graphical abstract

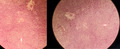

## 1 CASE PRESENTATION

A large flank mass was reported as a renal cell carcinoma of sarcomatoid variant. It had discordant clinical presentation; on further pathological work‐up with t(X;18), diagnosis of a synovial sarcoma was made.

**FIGURE 1:**
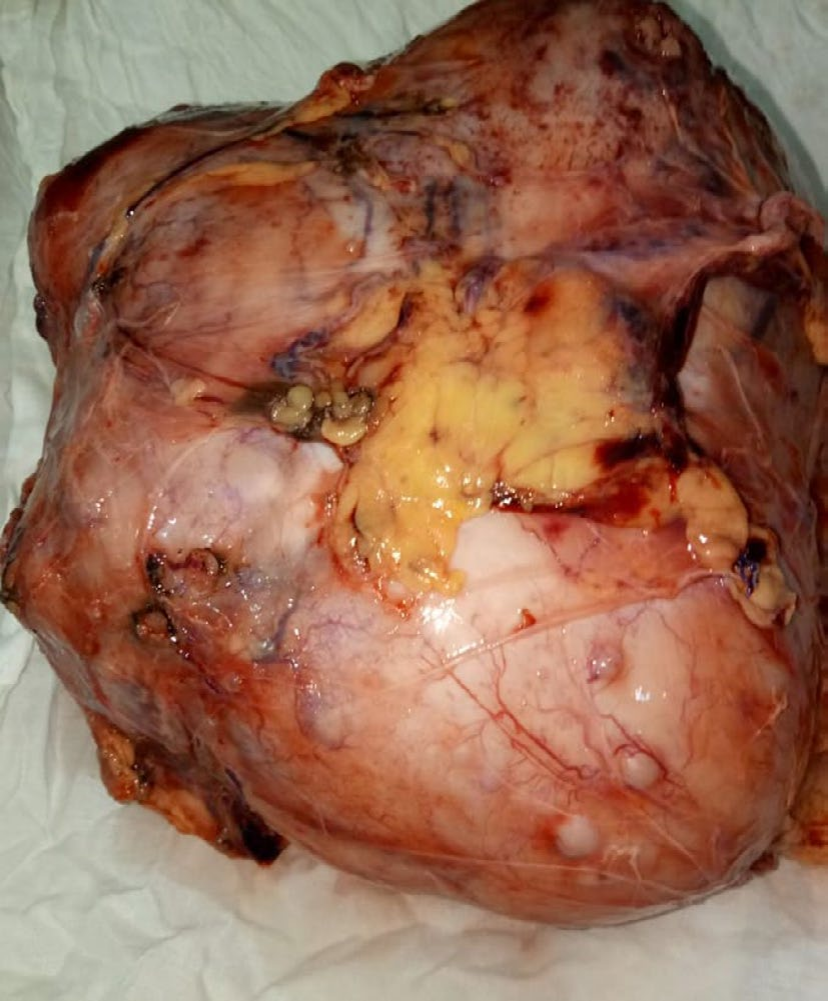
Sarcomatoid changes present on histopathology slide of renal mass showing renal cell carcinoma. Arrows point to areas of sarcomatoid change

Forty‐five‐year‐old female patient with right flank pain made multiple visits to rural general practitioners in Pakistan a year preceding presentation. Computed tomography scan reported “Massive heterogeneous mass replacing the whole of the right kidney, with residual scanty renal tissue, at its medial aspect.” During surgery a 15 × 18 cm mass involving the inferior vena cava was excised. (Figure 1).

Histopathology reported a 19 × 17 × 11 cm mass, involving the upper and lower pole adherent to the capsule with necrosis present. Lymph nodes free of tumor with positive markers: CK, Vimentin, TLE‐1, CD99, EMA, Cytokeratin AE1/AE3 and Cytokeratin 20.

**FIGURE 2:**
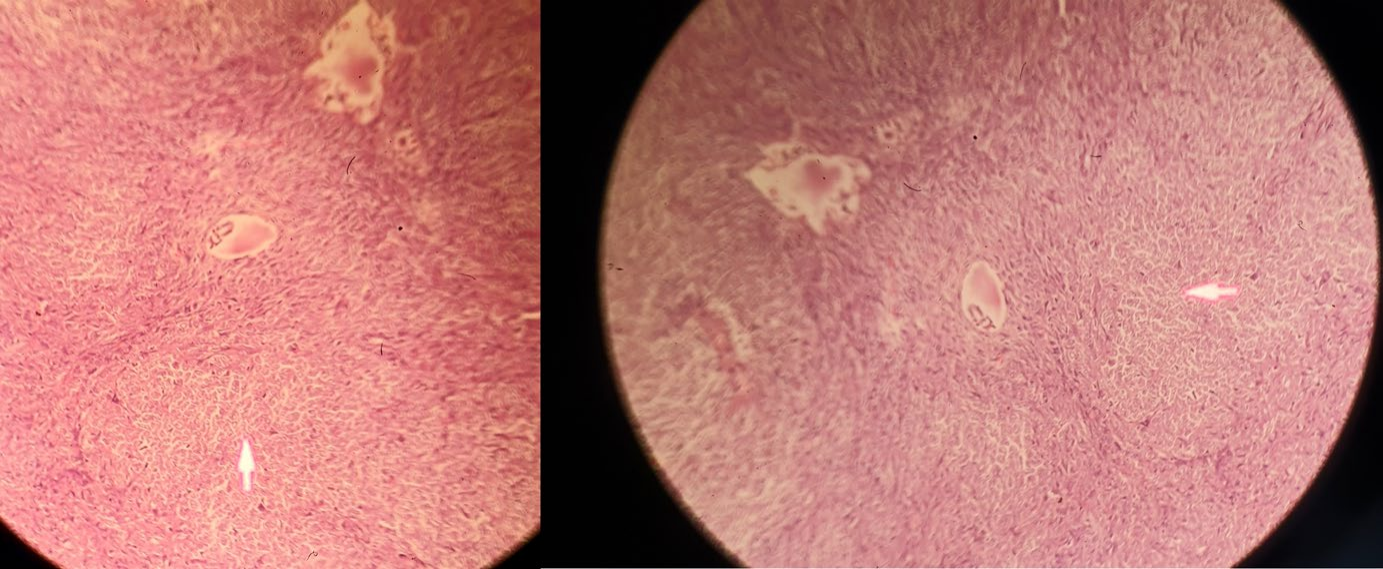
Surgical specimen of excised renal mass

Renal cell carcinoma, sarcomatoid variant was diagnosed, metastases were absent, and clinical presentation was discordant with reported aggressive tumor behavior. Chromosomal translocation (X;18) analysis returned positive and synovial sarcoma was diagnosed. (Figure 2).

Does sarcomatoid change in renal cell carcinoma merit analysis for synovial sarcoma?

## 2 DISCUSSION

Synovial sarcoma is extremely rare and aggressive, presenting in young adults commonly in periarticular tissue of the lower limbs. Multipotent stem cells lead to the sarcomatoid appearance.^1^ Renal sarcomas were differentiated from embryonal carcinoma by the characteristic translocation.^2^, ^3^ Case reports typically found large masses, grayish‐white or tan colored and with focal necrosis. Spindle cell morphology was consistently present.^4^, ^5^

## 3 CONCLUSION

For developing countries, where cytogenetic analysis is not routinely available in most public hospitals, endeavors to undertake t(X;18) analysis for sarcomatoid change in a renal cell carcinoma should be done.

## CONFLICT OF INTEREST

The authors have no conflict of interests to declare.

## AUTHOR CONTRIBUTIONS

Vishal Farid Raza: Was involved in data acquisition, analysis, manuscript drafting and revision, final approval, and is held responsible for content. Khalid Javeed Khan: Was involved in conceptual design, intellectual direction, analysis, manuscript drafting and revision, final approval, and is held responsible for content. Dawood Arshad: Was responsible for conceptual design, data acquisition and manuscript writing, editing and for analysis. Khalid Irshad: Was responsible for data acquisition, manuscript writing, editing and for data analysis.

## ETHICAL APPROVAL

All ethical considerations were done according to the Declaration of Helsinki and patient anonymity maintained.

## CONSENT

The authors have confirmed during submission that patient consent has been signed and collected in accordance with the journal's patient consent policy.

## Data Availability

The data that support this article are available from Pubmed.
