# Correction to “Knowledge of Cytogenetic Analysis for Synovial Sarcoma in Sarcomatoid Variant Renal Cell Carcinoma”

**DOI:** 10.1002/ccr3.70534

**Published:** 2025-05-27

V. F. Raza, D. Arshad, K. Irshad, and K. J. Khan, “Knowledge of Cytogenetic Analysis for Synovial Sarcoma in Sarcomatoid Variant Renal Cell Carcinoma,” Clinical Case Reports 9 (2021): e05052, https://doi.org/10.1002/ccr3.5052.

In the article above, an author's name was incorrectly published as Dawood Arshad. It should have been Muhammad Dawood Arshad.

The authors apologize for this error.

